# Emotional Dependence and Narcissism in Couple Relationships: Echo and Narcissus Syndrome

**DOI:** 10.3390/bs14121190

**Published:** 2024-12-13

**Authors:** María de la Villa Moral-Jiménez, Ava Mena-Baumann

**Affiliations:** Faculty of Psychology, University of Oviedo, 33003 Oviedo, Spain; uo283077@uniovi.es

**Keywords:** emotional dependence, narcissism, psychological abuse, romantic relationship

## Abstract

Emotional dependency consists of an extreme affective need that manifests pathologically, such that it has been established that the partners of emotionally dependent individuals exhibit narcissistic traits. The present study aimed to examine the relationship between emotional dependency, the narcissistic traits of a romantic partner, and psychological abuse, in addition to examining the sex differences in the first two variables. This study included 271 subjects (144 women and 127 men) between the ages of 18 and 66 (*M* = 36.9; *SD* = 14.748). This study compared individuals with and without emotional dependency, finding that those with emotional dependency reported more narcissistic partners. Furthermore, psychological abuse was positively correlated with the narcissism of the partner. There were no significant sex differences in emotional dependency or in the exhibition of narcissistic traits. The importance of examining the connections between emotional dependency, the narcissistic traits of a romantic partner, and the potential for psychological abuse within these relationships are highlighted.

## 1. Introduction

Romantic love is a multidimensional phenomenon that integrates not only emotional elements, but is also conceived as a sociocultural construction. From an evolutionary approach, it has been defined as a regulatory mechanism designed to solve interpersonal problems, giving it significant importance as a component of our adaptation [1]. The convergence of the multitude of aspects that mediate love makes it difficult, if not impossible, to define it without understanding the context in which it develops [2]. In this complex landscape of expressions of love, it is essential to explore the dynamics that can lead to dysfunctional patterns, such as those typical of emotional dependencies. Affective or sentimental dependencies are conceptualized as relational disorders characterized by the manifestation of a dependent attitude and addictive behaviors, in a context marked by role asymmetry [3]. Specifically, emotional dependence (ED) is defined as an extreme emotional need for pathological attachment that presents with craving, self-deception, possessiveness, emotional inescapability, an inability to break ties and negative feelings (guilt, emptiness, and fear of abandonment), among other descriptors [4]. The tendency to restrict a partner’s autonomy can lead emotionally dependent individuals to exhibit controlling, restrictive, and aggressive behaviors, highlighting their connection to proactive aggression [5].

There is no consensus regarding the origin of emotional dependence, although it has been proposed that there is a relationship between emotional dependence and the parenting styles experienced during childhood. Thus, it has been proven that boys and girls with emotional deficiencies [6] can present with emotional dependence in adulthood. Following this same line, a relationship has been found between emotional dependence and an anxious or preoccupied attachment style [7,8], and between ED and an avoidant–fearful attachment style [9]. A positive relationship has also been found between ED in romantic relationships and a pattern of unmet emotional needs from the maternal figure throughout childhood [10]. In contrast to the previous position, other authors have argued that emotional dependence is linked to a childhood characterized by an overprotective [11] and rigid [12] parenting style that results in the lack of an ability to deal with feelings of frustration and stress, creating in these individuals a deep fear of the loss of their close relationships in adulthood. 

As a whole, it has been determined that a realistic percentage of emotionally dependent people in Spain is 8.66% of the population [13]. However, studies like [14], focusing on young adults aged 18 to 30, have reported that 39.4% meet the criteria for emotional dependence, based on the established scale [4]. This figure is notably higher than the 24.6% found in those aged 16 to 31 [15], suggesting an increasing trend of dependence among younger generations, leading to more dependent lifestyles [4]. 

Adding to this profile, in the study of the relationship between sex and emotional dependence there have been contradictory results. On the one hand, several studies have not found any significant differences in relation to sex among those with ED [15,16,17]. In others, it has been concluded that men are more emotionally dependent than women [18,19], while a separate set of studies have highlighted the opposite, indicating that women show a greater tendency toward ED than men [20,21]. 

In relation to the type of partner chosen by those who are emotionally dependent, and the resulting dynamic created with said partner, they usually exhibit narcissistic tendencies towards which the emotionally dependent partner shows complacency. The partner chosen tends to be manipulative and exploitative, and believes they are superior to their partner and adopts a dominant position in the relationship, expecting a more submissive position from the other person. An analysis of this profile reveals many similarities with the characteristics of individuals who exhibit narcissistic traits or have narcissistic personality disorder. The tendency of people with ED to exhibit blindness towards their partner’s imperfections and their complacency towards their narcissism makes them perfect victims for the abuse and consequences of a relationship with a narcissistic person [22]. The partners chosen by emotionally dependent individuals are often characterized by strong self-esteem and an excessive, though distorted, self-confidence, which captivates the emotionally dependent. These partners are typically manipulative and exploitative, showing little empathy or affection. In summary, they assume a dominant role in the relationship, contrasting with the emotionally dependent partner’s more submissive position [3].

When defining narcissism, the distinction between pathological narcissism and narcissistic personality disorder (NPD) is crucial. The first is defined as maladaptive patterns of self-esteem regulation [23], and the second is defined in the *DSM-5* as a dominant pattern of grandiosity (both in fantasy and behavior), accompanied by a constant search for admiration and a significant decrease in the ability to experience empathy [24]. Specifically, it refers to a persistent pattern of grandiosity, a need for admiration, and a lack of empathy. This pattern is characterized by five or more of the following symptoms: (a) an exaggerated and unfounded sense of self-importance and talents (grandiosity); (b) a preoccupation with fantasies of unlimited success, influence, power, intelligence, beauty, or perfect love; (c) a belief in being special and unique, warranting association only with people of the highest status; (d) a need for unconditional admiration; (e) a sense of entitlement; (f) the exploitation of others to achieve personal goals; (g) a lack of empathy; (h) envy of others or the belief that others envy them; and (i) arrogance and haughtiness. Additionally, the symptoms must have begun in early adulthood [24].

Furthermore, an evaluation of epidemiological studies calculated a median prevalence of 1.6% in the general population [25], with higher rates observed in men [26,27]. This condition typically begins to develop in early adulthood. After an increase in the prevalence of NPD beginning in the 1960s [28,29], followed by a decline after the 2008 crisis and subsequent stabilization, rates were again documented at levels similar to those recorded in the 1980s and 1990s [30]. In that sense, narcissism has acquired great relevance in the consciousness of society, especially in the United States and on social media [31]. In various studies focused on examining the origins of NPD, it has been confirmed that it is influenced to an equal extent by both environmental factors and genetic factors [32,33]. Regarding the influence of the environment, the findings have varied widely. 

Furthermore, these manifestations can vary widely across different sociocultural contexts, so it is important to approach their generalization with caution. On the one hand, some authors have proven that developmental experiences, especially negative ones such as being rejected during childhood and a fragile ego during infancy, can contribute to the appearance of NPD in adulthood [34,35]. In contrast, it has been concluded that there is a direct relationship between parents’ overvaluation of their children’s abilities and their narcissistic traits [36,37].

Certain common characteristics of narcissistic people have been identified, such as their tendency to exhibit cognitive empathy while lacking emotional empathy [38], as demonstrated by neurobiological studies [39,40]. Likewise, NPD is linked to notions of superiority and a sense of entitlement to special treatment [41], in addition to a marked need for power [42]. These characteristics have previously been mentioned as distinctive of the partners of emotionally dependent people [3], especially the compatibility between the need for power of narcissists and the power games characteristic of the identity imbalances of people with ED. In relation to notions of superiority, narcissistic individuals often seek to impress others [43] by showcasing their talents, which can make them appear charismatic at first [44]. However, this initial impression can quickly shift, as their behavior may become perceived as belittling, confrontational, or insulting, depending on their own interests and needs [45,46]. 

Likewise, the effects of being in a close relationship with a narcissistic person have been investigated, revealing that the participants reported high levels of anxiety, depression, self-harm, and frustrated dependence patterns [47,48]. Additionally, patterns of abuse by narcissists have been frequently observed, with the findings indicating a significant positive correlation between narcissism and the perpetration of psychological violence in relationships [49]. A relationship between emotional dependence and psychological abuse has also been identified [50], drawing attention to the dysfunctionality of emotional ties that perpetuate the cycle of violence [51]. In such cases, the need for affection in emotionally dependent individuals can become so intense that behaviors suggestive of psychological violence may be downplayed to fulfill that emotional need. Consequently, a higher emotional dependence has been associated with increased psychological violence in couples [52]. This pattern also extends to controlling and surveillance behaviors in virtual environments [14]. 

After reviewing the characteristics of both the partners of emotionally dependent individuals and narcissistic individuals, a notable correspondence between the two profiles is evident. However, despite the existing research detailing these characteristics, and the suggestion of a relationship between emotional dependence and a partner’s narcissism [3], there is a gap in the literature regarding direct studies exploring this relationship within dysfunctional emotional dynamics. This gap in the research offers a valuable opportunity to explore and contribute to a relevant yet underexamined topic that could have significant implications for both theory and clinical practice.

Under the name of *Echo and Narcissus Syndrome*, we refer to the set of concurrent symptoms in a relationship between an emotionally dependent person, who maintains a dysfunctional attachment bond with psychological abuse, and their partner, who tends to exhibit the traits of a narcissistic personality, confronting an idealized love with love towards oneself.

Building on the above, the objective of this study is to examine the relationship between emotional dependence, narcissism, and psychological violence in couples, with the intention of providing data on the romantic relationships established between people with ED and those who present with narcissistic traits. Additionally, it is proposed to analyze the inter-sex differences in the emotional dependence and narcissistic traits of couples. Suffice it to remember that according to one of the myths collected by Ovid in The Metamorphosis, the young nymph Echo was condemned by Hera, the wife of Zeus, to repeat the end of the phrases she heard so that talkative and happy young woman became silent and isolated. The encounter with Narcissus and his splendid beauty led her to fall in love with him, to chase him through the forest near a stream, afraid of being seen, and to idealize his person. On one of the many occasions when she watched him, a noise she made when stepping on a branch alerted Narcissus to her presence. Echo only found Narcissus’ mockery and was consumed with pain repeating, as if in a whisper, the last words she had heard from him, "how stupid... how stupid... what... stupid... ask". For his part, Narcissus suffered the punishment of the goddess Nemesis and, upon seeing his image reflected in the river, he fell in love with himself and died of starvation as he was eternally enthralled with his own contemplation.

With the proposed objectives, the following hypotheses are presented: (H1) It is expected that the partners of people who are categorized as emotionally dependent will present with narcissistic traits to a greater extent than the partners of people who are not emotionally dependent. (H2) It is assumed that there will be a positive correlation between the partner’s narcissism and the psychological violence experienced by the person with ED. (H3) It is predicted that there will be no statistically significant inter-sex differences in emotional dependence. (H4) It is predicted that there will be statistically significant inter-sex differences in the narcissistic traits of the partners. (H5) It is hypothesized that the relationship between emotional dependence (ED) and perceived psychological abuse will be partially mediated by the narcissistic personality traits of the partner (NTRP). (H6) It is assumed that gender does not moderate the relationships between ED, the partner’s narcissism (NTRP), and the perceived psychological abuse, meaning these relationships will be consistent across the male and female participants. (H7) It is predicted that gender does not moderate the relationship between emotional dependence (ED) and the narcissistic traits of romantic partners (NTRP).

## 2. Materials and Methods

### 2.1. Participants 

A total of 271 subjects participated in this study, providing information about themselves and their partners. The participants’ ages ranged from 18 to 66 years, resulting in a mean age of 36.9 (SD = 14.748). The couples also had an age range of 18 to 66 years, with a mean age of 36.70 (SD = 14.510). The sample included 144 women (53.1%) and 127 men (46.9%). The sex of their partners was similarly distributed, with 135 women (49.8%) and 136 men (50.2%). 

As for educational background, 144 had completed a University Degree or Higher (53.1%); 111 had completed a Baccalaureate or an Intermediate Degree (41%); 14 had completed Compulsory Secondary Education or a Basic Degree (5.2%); and finally, 2 had completed Primary Education (0.7%). Of their partners, 140 had completed a University Degree or Higher (51.7%); 97 had finished a Baccalaureate or an Intermediate Degree (35.8%); 27 had completed Compulsory Secondary Education or a Basic Degree (10%); and 7 had completed Primary Education (2.6%).

Since the object of study was ED and narcissism in relationships, the essential criterion for participating was to currently have a partner or have had one in the past. Of the 271 participants, 207 were currently with their partner (76.4%), of which 141 (52%) indicated that “we are together” and 66 (24.4%) shared that “we are married”. The remaining 64 people (23.6%) had been in a relationship in the past; 55 (20.3%) selected “we were together, but now we are not” and 9 (3.3%) selected “we were married, but now we are divorced”.

Finally, information on the duration of the said relationships was also collected. Thus, 94 people (34.7%) indicated a duration of “+10 years”; 46 (17%) indicated a duration of “less than 1 year”; 42 (15.5%) shared that the relationship lasted or has lasted “2 years or more”; 33 (12.2%) indicated that the duration of the relationship is or was “5 to 10 years”; 32 (11.8%) revealed a duration of “3 to 5 years”; and finally, the remaining 24 people indicated that their relationship has extended or did extend for “1 year or more”.

### 2.2. Measuring Instruments

Three main constructs or variables were analyzed in the preparation of this study: emotional dependence, the narcissistic traits of partners, and psychological violence, through an instrument made up of three scales that will be described below. It should be noted that the sociodemographic data of the participants were also collected. These included the gender, age, educational level, place of residence, current relationship status, and duration of the relationship.

Affective or emotional dependence scale of the Inventory of Interpersonal Relationships and Sentiment Dependencies (IRIDS-100) [53].

The IRIDS-100 focuses on evaluating various forms of relational dependence, including emotional or affective dependence, codependency, and bidependence. Within this context, the Affective Dependency Scale was applied, composed of 24 items, whose responses are rated on a 5-point Likert scale, ranging from “strongly disagree” to “strongly agree”. A higher score on the scale indicates a greater inclination towards emotional dependence, and a threshold of 2.45 points is established as identifying the presence of ED in the individual evaluated. During the validation of the scale for the Spanish population [53], a Cronbach’s alpha coefficient of 0.971 was achieved for a sample of 880 subjects. In this analysis, a reliability coefficient value of 0.880 was obtained for the emotional dependency scale.

Inventory to evaluate Psychological Abuse in Couple Relationships (IAPRP) [54].

The IAPRP is designed to assess psychological abuse in relationships. It is a brief inventory, made up of 17 items, that evaluates different types of emotional abuse, such as withholding information, controlling activities, and prohibiting things, including the use of money or transportation. The participants had to indicate the frequency with which the different types of abuse detailed in each item occurred during the last year of the relationship. They had five options for this, from “never happened” to “more than 10 times”. “per year”. The inventory has a very high reliability; a Cronbach’s alpha of 0.99 was obtained. In the present work, an alpha value of 0.858 was reached.

Narcissistic Personality Inventory (NPI)—Spanish Version [55].

The NPI, originally developed by [56], has become the most widely validated and used instrument in empirical research on narcissism in general populations, allowing for the exploration of individual differences in this personality trait. It has 40 items, each made up of two statements, from which you must select the one that best suits your personality. Obtaining a higher score indicates a greater presence of narcissistic traits, and because it only measures the degree of the presence of narcissistic traits, there is no cut-off point in the score from which it could be determined that someone is narcissistic. In its validation for the Spanish population, a reliability coefficient of 0.72 was reported. For the present work, the format of the scale was adapted from being a self-report to being completed by the partner of the person whose narcissistic traits we intended to measure, to facilitate the collection of responses and ensure reaching a greater number of participants. In this study, with this adapted version, a reliability level of 0.888 was reached.

### 2.3. Procedure

The questionnaire was created using the Google Forms platform and distributed via social networks for electronic completion. The sampling was a non-probabilistic, snowball type. The data were collected anonymously and exclusively for research purposes, as indicated to the participants at the beginning of the form, in accordance with the ethical standards of the American Psychological Association [57]. Additionally, the explicit consent of the respondents was required to collect the data, checking a box to communicate their agreement, in accordance with Organic Law 3/2018 [58]. 

### 2.4. Design 

It is an ex post facto and non-experimental design, since the variables were not deliberately manipulated, and are descriptive and correlational. Furthermore, it is a cross-sectional, empirical, and quantitative study [59].

### 2.5. Data Analysis 

To carry out the data analysis, statistical software SPSS, version 27, and the JASP 0.9 program were used. To check the normality of the data, a Kolmogorov–Smirnov test was used. Despite the variances being homogeneous, the assumption of normality was not met by Levene’s test, which was why non-parametric tests were used for the subsequent hypothesis contrasts.

To test the first hypothesis, a contrast of means for the independent samples was carried out. For this, a Mann–Whitney U test was used, since the assumption of normality was not met. The group with emotional dependence and the group without emotional dependence were compared for the variables related to the narcissistic traits of their partners. In the case of the second hypothesis, a Spearman correlation test was performed between the indicators “narcissistic traits of the romantic partner” and “psychological abuse suffered by the subject in the romantic relationship”. To analyze the differences in emotional dependence based on sex, a difference in proportions was applied and, in the case of the variables being the narcissistic traits of the romantic partner and the sex of said partner, a Mann–Whitney U test was applied. In testing the fifth hypothesis, a mediation analysis was conducted to examine whether narcissistic personality traits (NPDs) mediated the relationship between emotional dependence (ED) and perceived psychological abuse. Given that the data did not meet normality assumptions (Shapiro–Wilk tests, *p* < 0.001), bootstrapping was employed to ensure robust estimates; this provided estimates of the indirect, direct, and total effects with 95% confidence intervals. Bootstrapping, a non-parametric resampling method, generates confidence intervals based on repeated sampling (5000 resamples) and does not rely on parametric assumptions. The mediation analysis included testing the direct effect of ED on psychological abuse (total effect), assessing the indirect effect of ED on psychological abuse via NPDs, and calculating the proportion of the total effect mediated by NPDs. To test Hypothesis 6, a moderation analysis was conducted to examine whether gender moderates the relationships between emotional dependence (ED), narcissistic personality traits (NTRPs), and psychological abuse. A hierarchical regression approach was used. In the first step, the main effects of emotional dependence, narcissistic personality traits, and gender (G) were included. In the second step, interaction terms (G × ED and G × NTRPs) were added to the model. Variance Inflation Factors (VIFs) were calculated to ensure the absence of multicollinearity among the predictors, and Cook’s Distance was employed to identify potential influential data points. Robust standard errors were applied to address heteroscedasticity in the data. For the contrast in the last hypothesis, to test whether gender moderates the relationship between emotional dependence (ED) and the narcissistic traits of romantic partners (NTRPs), a moderation analysis was conducted. Emotional dependence was included as the independent variable, the narcissistic traits as the dependent variable, and gender (G) as the moderator. The analysis involved creating an interaction term (ED × G), which was included in the regression model. Variance Inflation Factors (VIFs) were calculated to ensure no multicollinearity among the predictors, and Cook’s Distance was employed to identify potential influential data points. To address heteroscedasticity in the data, robust standard errors (HC3) were applied to ensure valid inference. The model examined both the main effects of ED and gender, as well as their interaction effect.

## 3. Results

Firstly, according to the established scale on emotional dependence [4], 209 people scored within the “Absent” level (77.1%), with 22.9% (*n* = 62) of the participants reporting emotional dependence. 

### Emotional Dependence and Narcissistic Traits of Romantic Partners

Once classified, the two groups were analyzed based on the variable narcissistic traits of their romantic partners. For this analysis, the Mann–Whitney U statistic was used. It was found that the emotionally dependent subjects scored higher (*M* = 17.935; *SD* = 8.398) regarding the narcissistic traits of their partners than the subjects without emotional dependence (*M* = 14.550; *SD* = 7.744). These differences were significant (*ρ* = 0.005), with a medium effect size, since it was greater than 0.2 (rbis = −0.234) (see Table 1).

Secondly, the Spearman correlation was used to analyze the relationship between the psychological abuse suffered by the subject in a romantic relationship and the narcissistic traits of their partner (Table 2). This coefficient measures the strength and direction of the association between two ordinal variables, providing a robust perspective on non-linear relationships. It was found that psychological abuse correlates positively with the narcissistic traits of the romantic partner (ρ < 0.001). This relationship is considered low since 0.2 < rho < 0.39 (rho = 0.325). This implies that as the narcissistic traits of a partner increase, so does the frequency of perceived psychological abuse. Although the coefficient is low, its statistical significance indicates that the relationship is consistent within the analyzed sample. In this case, 10.5% of the psychological abuse that the subjects suffered in their romantic relationships could be associated with the narcissistic traits of their partners. The analysis of the average values reinforces the previously noted trend. To explore the relationship between the two variables, the sample was divided into two groups based on the percentiles of the narcissistic personality inventory (NPI). The participants who reported higher levels of perceived narcissism in their partners (≥75th percentile) also reported a higher average of psychological abuse (M = 9.96, SD = 10.30) compared to those who reported lower levels of perceived narcissism (≤25th percentile, M = 7.40, SD = 8.19). 

Regarding the third hypothesis, a difference in proportions test was used for two independent samples (Table 3), and it was concluded that there is no statistically significant difference in the proportion of emotional dependence among women and men based on the results of the normal approximation test (*Z* = −0.27, *ρ* = 0.785). Furthermore, the 95% confidence interval for the difference in proportions [−0.114, 0.086] includes zero, which also indicates that the observed difference is not significant.

Next, to analyze whether there was a relationship between the narcissistic traits of the couples and their sex, the Mann–Whitney U statistic was used again (Table 4). No statistically significant differences were found between the narcissistic traits of the female partners (M = 15.104; SD = 7.940) and those of the male partners (M = 15.544; SD = 8.102).

To test the fifth hypothesis, a mediation analysis was conducted with emotional dependence (ED) as the predictor, the narcissistic traits of the romantic partner (NTRP) as the mediator, and psychological abuse as the outcome. Bootstrapping (5000 resamples) was used to account for the non-normal distribution of the data. The results indicate that the indirect effect of ED on psychological abuse through the NTRP is significant (*B* = 0.184, 95% *CI* = [0.104, 0.276]), supporting the mediating role of the NTRP. The direct effect of ED on psychological abuse (controlling for the NTRP) is also significant (*B* = 0.295, 95% *CI* = [0.192, 0.400]). Finally, the total effect of ED on psychological abuse is significant (*B* = 0.480, 95% *CI* = [0.360, 0.607]). Approximately 38.44% of the total effect of ED on psychological abuse is mediated by the NTRP, highlighting its critical role in the dynamics of dysfunctional relationships (Table 5).

To evaluate whether gender moderates the relationships between emotional dependence (ED), narcissistic personality traits (NTRPs), and psychological abuse, a moderation analysis was conducted (Table 6). The findings indicate that the main effects of emotional dependence (*B* = 0.452, *p* < 0.001) and narcissistic traits (*B* = 0.715, *p* < 0.001) on psychological abuse are significant. However, gender is not a significant predictor (*B* = 0.232, *p* = 0.271), nor are the interaction terms (gender × ED: *B* = −0.063, *p* = 0.542; gender × NTRPs: *B* = −0.273, *p* = 0.233). These results suggest that the relationships between emotional dependence, the narcissistic traits of the romantic partner, and psychological abuse are consistent across genders.

A moderation analysis was conducted to evaluate whether gender moderates the relationship between emotional dependence (ED) and the narcissistic traits of the romantic partner (NTRP). The results show that the interaction term ED × G is not statistically significant (B = 0.047, *p* = 0.360), indicating that the relationship between ED and the NTRP does not differ based on gender. Additionally, neither the main effect of ED (B = −0.008, *p* = 0.820) nor the main effect of gender (B = −0.584, *p* = 0.243) is statistically significant (Table 7). The maximum Cook’s Distance is 0.075, suggesting no influential data points, and the VIF values indicate no multicollinearity among the predictors. These findings suggest that the association between ED and the NTRP is consistent across genders, aligning with the hypothesis that gender does not moderate this relationship.

## 4. Discussion

Certainly, the dysfunctional relationships that emotional dependents maintain with their partners exhibit unique characteristics during which they undergo processes of submission and/or subjugation, involvement in power games, dominance, surveillance and control, and even psychological violence. Based on these findings, it can be concluded that emotionally dependent individuals are more likely to maintain romantic relationships with partners who exhibit narcissistic traits than those who are not emotionally dependent, as predicted in the first hypothesis. This aligns with the previous literature on emotional dependence [3], which highlights how emotional dependents tend to be complacent toward their partners’ narcissism. Narcissistic partners often exploit the idealization that emotional dependents experience, as well as their difficulty in breaking ties, showing manipulative and exploitative tendencies. This theory has also been defended by other authors [22], which makes people with ED the perfect victims of the abuse associated with maintaining a romantic relationship with a narcissistic person.

Additionally, another factor that may explain why people with ED are more likely to enter into romantic relationships with narcissistic partners is that the characteristics and behaviors that emotionally dependent people exhibit in this type of relationship, such as their differential perception of the affective–dependent relationship and its characteristic cognitive profile with cognitive distortions and coping strategies [60], represent an added difficulty in getting out of them, despite how destructive they are [61]. Understanding the underlying factors that contribute to their inability to leave a relationship could help develop more effective support strategies and therapeutic approaches.

A positive and significant relationship is confirmed between the psychological abuse reported by the subjects and the narcissistic traits exhibited by their partners, and it is concluded that 10% of the reported psychological abuse is linked to the narcissism of the romantic partner. These results are similar to those obtained in other studies, in which a positive relationship between narcissism and psychological abuse within romantic relationships has been reported [62]. In this sense, important contributions have been made to the study of the interpersonal dysfunction characteristics of narcissistic people, the abuse they perpetrate, and the psychological consequences it has for the recipients of this type of behavior [47,48,63].

Regarding inter-sex differences, no differential profile was found among emotional dependents, so the results are in line with those that have been obtained by other authors [15,16,17]. However, these findings contradict, on one hand, the results that have indicated that women are more likely to have ED [11] and, on the other hand, the trend that has been observed in men [18,19,21]. Finally, regarding the analysis of inter-sex differences in the presentation of narcissistic traits, no significant differences were observed, contradicting the popular conception that narcissism is more prevalent in men and differing from the findings obtained in previous research [27]. It has been theorized that these differences may stem from limitations in the conceptualization and measurement of narcissism, which tends to focus more on masculine traits, while the typical characteristics of narcissistic women are often overlooked [64,65]. 

The mediation analysis highlighted the dynamic mechanisms underlying the relationship between emotional dependence (ED), narcissistic personality traits (NPDs), and psychological abuse. The results demonstrate that while ED directly influences psychological abuse, a significant portion of this effect (38.44%) is mediated by NPDs. This finding underscores the role of NPDs as a critical mechanism through which ED exacerbates dysfunctional relationship dynamics. These results extend previous theoretical perspectives by illustrating how emotionally dependent individuals’ vulnerability to abusive behaviors is amplified in relationships with partners exhibiting narcissistic traits. The results are consistent with the broader literature, which has emphasized the aggressor’s personality traits in shaping the dynamics of power and control within abusive relationships [47,48]. 

The results of the moderation analysis reveal that gender does not moderate the relationships between emotional dependence, the narcissistic traits of the romantic partner, and psychological abuse. These findings suggest that the dynamics between these variables are consistent across the male and female participants. While gender may influence other aspects of abusive relationships, the current results indicate that the predictive effects of ED and the NTRP on psychological abuse are not contingent upon the participant’s gender. 

Finally, the results of the second moderation analysis indicate that gender does not moderate the relationship between emotional dependence (ED) and the partner’s narcissistic traits (NTRPs). This finding supports the hypothesis that the dynamics between ED and the NTRPs are consistent across the male and female participants. These observations align with broader theoretical frameworks suggesting universal mechanisms underpinning emotional dependence and its associated dynamics in romantic relationships [3,22]. 

### Limitations and Future Lines of Research

As expected, a series of limitations have been identified in this study. Firstly, the sample used is not very large; this raises the possibility of representativeness issues within the population, which could hinder the ability to extrapolate the results. Additionally, when using non-probability snowball sampling, selection biases are possible. Another important limitation is the cross-sectional design of this study. Although this design allowed us to analyze the relationships among the variables at a specific time, it was not possible to establish causal relationships; hence, it would be beneficial to consider a longitudinal design in future research. Furthermore, while the mediation analysis provided valuable insights into the dynamics between emotional dependence (ED), narcissistic personality traits (NPDs), and psychological abuse, the data did not meet normality assumptions, as confirmed by the Shapiro–Wilk tests. To address this, a bootstrapping approach with 5000 resamples was employed, ensuring robust estimates of the indirect, direct, and total effects. While this method is statistically sound and widely accepted for non-normal data, it introduces additional complexity that may not fully capture the nuances in the relationships. Taking into account the subject matter under study, it is advisable to be cautious in the interpretation of the results given the possible action of social desirability bias. Another limitation is the format of the scale used to measure the narcissistic traits of the romantic partners. Since no validated scale was available to measure narcissistic traits in the Spanish population that could be completed by an observer, the NPI was adapted to assess the subjects’ perceptions of the narcissistic traits exhibited by their partners. Methodological limitations may arise from adapting the content of the items to the evaluation of couple relationships, and we suggest the development of more suitable measurement tools for the indirect assessment of narcissistic traits.

As future lines of research, it would be of interest to continue exploring the interaction between emotional dependence (ED) and narcissistic traits in romantic relationships, integrating such knowledge into clinical interventions. Furthermore, through additional research on the motivations that lead people with ED to remain in these relationships, valuable information could be provided to be applied to psychotherapeutic objectives for the comprehensive treatment of emotional dependence [66,67]. A prior multifrontal evaluation and an intervention proposal based on cognitive–behavioral models have been proposed, both for individual and group psychotherapy, as well as training strategies for psychosocial skills. Designing and implementing individualized therapeutic strategies that address the well-established link between narcissism and psychological abuse is particularly important. These strategies could help victims recognize behaviors, such as devaluation, indifference, blame shifting, and hostility, often exhibited by emotionally exploitative partners. Further exploring the specific dynamics between narcissists and their romantic partners who have experienced psychological abuse may be beneficial for clinical professionals [68,69].

## 5. Conclusions

In conclusion, the findings of this study suggest a significant positive association between emotional dependence and the perception of narcissistic traits in romantic partners, indicating that individuals with emotional dependence are more likely to engage in relationships with such partners. Additionally, a positive and significant relationship is identified between participants’ reported psychological abuse and their romantic partners’ narcissistic traits. From all of this, multiple implications arise at the clinical and psychosocial levels, since it would be of interest to delve deeper into the study of relationships of control and abuse of power, as well as the processes of submission and subjugation that unfold in these dysfunctional relationships and that perpetuate the cycle of violence in couples. 

## Figures and Tables

**Table 1:** Contrast of means for people with and without emotional dependence as function of narcissistic traits of their romantic partners.

	ED	N	Mdn	M	DT	CV	U M–W	*p*	Effect Magnitude (rbis)
NTRP	With DE	62	18	17.935	8.398	0.468	4962	ρ = 0.005	−0.234
	Without DE	209	14	14.550	7.744	0.532			

Note: ED = emotional dependence; N = sample size; Mdn = median; M = mean; DT = standard deviation; CV = coefficient of variation; U M-W = Mann–Whitney U statistic; ρ = significance; rbis = Rank Biserial Correlation; NTPR = narcissistic traits of the romantic partner.

**Table 2:** Spearman correlation between the variable “psychological abuse suffered by the subject in the relationship” and the variable “narcissistic traits of their romantic partner”.

	Narcissistic Traits of the Romantic Partner	
**Psychological Abuse**	Spearman’s Rho	Signification (ρ)
	0.325	<0.001

**Table 3:** Inter-gender differences in proportions of emotional dependence.

	G	*N*	Method	*Z*	*p*	CI
ED	Female	144	Norma approach	−0.27	*ρ* = 0.785	(−0.114338; 0.086342)
	Male	127	Fisher’s exact		*ρ* = 0.885	

Note: ED = emotional dependency; G = gender of romantic partner; N = sample size; Z = Z value; ρ = significance; CI = 95% confidence interval for the difference.

**Table 4:** The contrast of means for the narcissistic traits of couples according to their gender.

	G	N	Mdn	M	DT	CV	U M-W	*p*
NTRP	Female	135	14	15.104	7.940	0.526	8982.500	0.760
	Male	136	14	15.544	8.102	0.521		

Note: G = gender of romantic partner; N = sample size; Mdn = median; M = mean; DT = standard deviation; CV = coefficient of variation; U M-W = Mann–Whitney U statistic; ρ = significance; NTRP = narcissistic traits of the romantic partner.

**Table 5:** The results of the mediation analysis predicting psychological abuse from emotional dependence and the narcissistic traits of the romantic partner.

Effect Type	Predictor (IV)	Mediator (M)	Outcome (DV)	Estimate (B)	CI	Proportion Mediated (%)
Indirect Effect	ED	NTRP	PA	0.184	[0.104, 0.276]	38.44
Direct Effect	ED		PA	0.295	[0.192, 0.400]	
Total Effect	ED		PA	0.480	[0.360, 0.607]	

Note: B = unstandardized regression coefficient; CI = 95% confidence interval; ED = emotional dependence; NTRP = narcissistic traits of the romantic partner; PA = psychological abuse.

**Table 6:** The results of the moderation analysis predicting psychological abuse, testing gender’s role as a moderator.

Predictor Variable	*B*	RSE	*t*	*p*	CI
ED	0.452	0.063	7.12	<0.001	[0.328, 0.576]
NTRP	0.715	0.160	4.47	<0.001	[0.404, 1.026]
G	0.232	0.213	1.10	0.271	[−0.183, 0.647]
ED × G	−0.063	0.102	−0.61	0.542	[−0.261, 0.135]
NTRP × G	−0.273	0.227	−1.20	0.233	[−0.725, 0.179]

Note. B = unstandardized regression coefficient; RSE = robust standard error; *t* = test statistic for the regression coefficient; *p* = probability value for the test statistic; CI = 95% confidence interval; ED = emotional dependence; NTRP = narcissistic traits of the romantic partner; G = gender.

**Table 7:** The results of the moderation analysis predicting narcissistic traits in romantic partners based on emotional dependence, testing gender’s role as a moderator.

Predictor Variable	*B*	RSE	*t*	*p*	CI
ED Total	−0.008	0.036	−0.228	0.820	[−0.080, 0.063]
G	−0.584	0.499	−1.170	0.243	[−1.565, 0.398]
ED x G	0.047	0.051	0.917	0.360	[−0.054, 0.148]

*B* = unstandardized regression coefficient; RSE = robust standard error; *t* = test statistic for the regression coefficient; *p* = probability value for the test statistic; CI = 95% confidence interval; ED = emotional dependence; G = gender.

## Data Availability

The data are contained within the article and the recordings and raw datasets supporting the conclusions of this study will be made available by the corresponding author on request.
